# Supplementary material for: In vivo ultrasound-switchable fluorescence imaging
Source: Sci Rep. 2019 Jul 8;9:9855. doi: 10.1038/s41598-019-46298-2 (PMC6614554; doi:10.1038/s41598-019-46298-2)
Supplement: Supplementary file 1 — Supplementary information [file 41598_2019_46298_MOESM1_ESM.pdf]

Supplementary Information

In vivo ultrasound-switchable fluorescence imaging

Tingfeng Yao, [1,2*] **Shuai Yu,** [1,2*] **Yang Liu**[1,2] **and Baohong Yuan**[1,2+]

[1]Ultrasound and Optical Imaging Laboratory, Department of Bioengineering, University of Texas at Arlington, Arlington, TX 76010, USA

[2]Joint Biomedical Engineering Program, University of Texas at Arlington and University of Texas Southwestern Medical Center at Dallas, Dallas, TX 75235, USA

[+]Correspondence and requests for materials should be addressed to B.Y. (baohong@uta.edu)

* These authors contributed equally to this work.

Switching property of the ICG-NPs. The method measuring the fluorescence intensity of the ICG-NPs as a function of temperature was described in our previous study[1]. As shown in Figure S1, the LCST of the ICG-NPs used for tissue phantom experiments was ~24─25 °C, and for *in vivo* experiments, it was ~35─36 °C.

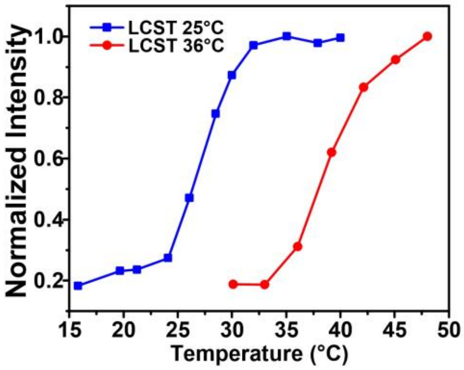

Figure S1. Normalized fluorescence intensity of the ICG-NPs as a function of temperature. (ICG-NPs with a LCST of 24-25 °C: blue solid line with square; ICG-NPs with a LCST of 35-36 °C: red solid line with circle)

Size and concentration of the ICG-NPs. To measure the size of the ICG-NPs, a 50 μL ICG-NPs sample with an LCST of ~35─36 °C was diluted with 3 mL deionized water. A dynamic light scattering system (ZetaPALS, Brookhaven Instruments Corporation, NY, USA) was used to measure the hydrodynamic size of nanoparticles at 25 °C. The result shows that the mean and median hydrodynamic diameters of the nanoparticles were 359.2 nm and 334.8 nm, respectively. The polydispersity was 0.151.

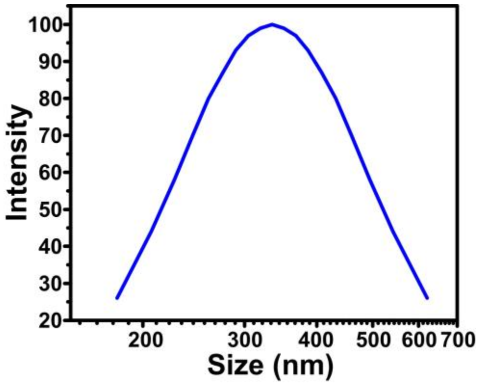

Figure S2. Dynamic light scattering of the synthesized ICG-NPs.

To measure the concentration of the ICG-NPs, a freeze dryer (FreeZone, Labconco, Kansas City, MO, USA) was used to freeze dry 15 mL nanoparticles solution with a vacuum at 0.01 mBar and a temperature of -50 ˚C. Afterwards, the obtained sample was weighed. The concentration of the nanoparticles was calculated by using the following equation:

$$Nanoparticle\ Concentration\ (\frac{mg}{ml}) = \frac{Mass\ of\ the\ sample\ after\ freeze\ dry\ (mg)}{Volume\ of\ the\ sample\ used\ to\ freeze\ dry\ (ml)}$$

which gave a result of 24.60 mg/mL.

Measuring the temperature increase in silicone phantom and porcine heart tissue. Both the silicone phantom and porcine heart tissue had a thickness of 7 mm and were placed on the parafilm (PM-992, BEMIS Company Inc., Neenah, WI, USA). The gap between the lower surface and parafilm was filled with ultrasound gel (01-08, Aquasonic 100, Parker Laboratories Inc., Fairfield, NJ, USA), while the phantom's upper surface was exposed to air. The high intensity focused ultrasound transducer (HIFU, 2.5 MHz, H-108, Sonic Concepts Inc., Bothell, Washington, USA) was positioned under the phantom, but the ultrasound wave was focused on the upper surface of the phantom. An infrared camera (FLIR A300, FLIR Systems, Wilsonville, Oregon, USA) with a frame rate of 20 Hz was positioned on the top of the phantom and was used to record the temperature distribution on the upper surface of the phantom. The ultrasound exposure time was 400 ms under a driving voltage of 200 mV (~4.82W ultrasound power). As an example, the left panel of Figure S3(a) shows the temperature dynamic variation after HIFU exposure at the central position where temperature is spatially maximum. The right panel of Figure S3(a) shows the 2D temperature distribution on the silicone phantom's upper surface at the time when the peak temperature was reached. Figure S3(b) shows the corresponding results in the porcine heart tissue. The temperature increases quickly during the ultrasound exposure and decays slowly after the exposure. The absolute values of the peak temperature in the two phantoms are ~68.64 and ~29.84 °C, respectively. The HIFU-induced temperature rise is much more in the silicone phantom (~43.30 °C,) than in the heart tissue (~5.30 °C) with the same driving voltage. This is due to the larger ultrasound absorption coefficient of the silicone. Figure S4 shows the HIFU-induced temperature rise in the porcine heart tissue as a function of the driving voltage. The measurement method is the same as the one described in Figure S3. Obviously, raising the driving voltage increases the temperature rise. A superlinear relationship between the driving voltage and the temperature rise can be observed. During USF imaging, it will be difficult to quantify the temperature rise because the IR camera cannot detect temperature inside the tissue. However, the above method and results can provide a rough guideline about the relationship. In this study, to avoid potential thermal damage to the tissue, the temperature rise was limited to a few Celsius degrees by controlling the driving voltage.

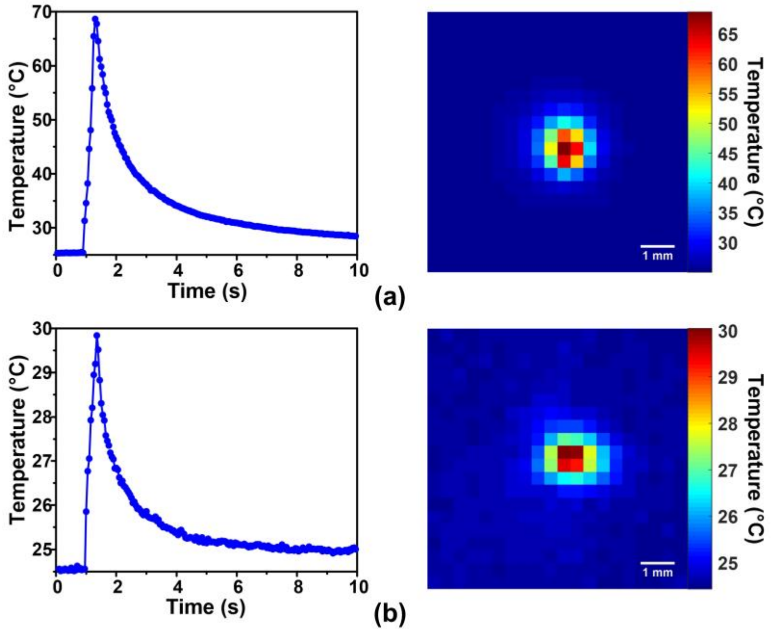

Figure S3. Comparison of the temperature rise with 200 mV driving voltage in (a) a silicone phantom and (b) a porcine heart tissue.

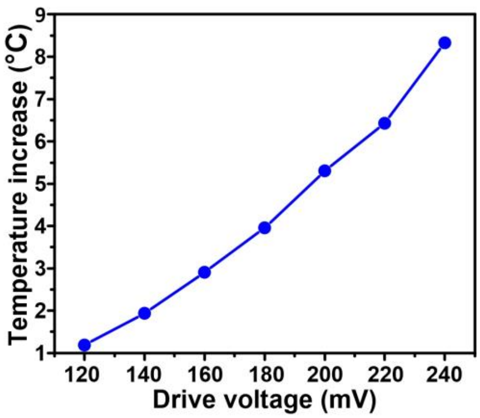

Figure S4. Temperature rise in the porcine heart tissue as a function of driving voltage.

USF image processing. Figure S5 shown here is based on the experimental data of the *in vivo* USF imaging of the mouse spleen. Figure S5(a) shows the raw USF signal output from the lock-in amplifier (the blue line). The oscillation is caused by the mouse breath. By fitting the raw USF signal via a polynomial fitting, the USF signal is smoothed (the red line). After fitting, the USF signal strength, defined as the maximum value of the fitted signal in the range of 2 to 5s, can be acquired for each location. A USF image can be formed after a 2D or 3D scanning. As an example, Figure S5(c) shows a 2D image representing the USF signal strength distribution and therefore the distribution of the USF contrast agents on the XY plane.

To remove some obvious noises or artifacts, in each experiment, the shapes of all the fitted USF data were evaluated, and three typical fitted USF signals were selected as the references (Figure S5(b)). Thus, each fitted USF signal was correlated with the three references, and three correlation coefficients were calculated for each fitted USF signal at each location. In this study, based on our experience and observation, we defined noise as none of the three correlation coefficients being greater than 0.8. In other words, to consider a signal as a USF signal, at least one of the three correlation coefficients had to be greater than 0.8. The data in Figure S5(c) were processed using this correlation method, and the result is shown in Figure S5(d). Clearly, some artifacts caused by noise and mouse breathing were removed by using this correlation method.

After correlation, a 3D USF image could be obtained by combining the multiple layers of the 2D USF images (XY plane). By 3D interpolation, the pixel size of the USF image was reduced to 50.8 μm instead of 762 μm (Figure S5(e)). Afterwards, the interpolated 3D USF image was divided into 2D images (YZ plane) used for image segmentation. By segmenting the 2D image that had the maximum intensity value among all the images (YZ plane) using 2D Otsu method[2], a threshold was obtained. All the 2D USF images (YZ plane) were then segmented by applying this threshold. As shown in Figure S5(f), some background signals shown in Figure S5(e) were removed after the segmentation.

Strictly speaking, the USF signal strength is not in direct proportion to the quantity of contrast agents along the axial direction because of the optical and acoustic attenuation. To compensate for this attenuation, the models describing photon diffusion and acoustic attenuation may be adopted. However, we did not consider these methods for the following reasons: (1) in order to adopt those models many background parameters of the tissue will be needed, such as optical absorption and scattering coefficients and the acoustic attenuation coefficient, which are usually unknown and will make the imaging too complicated; (2) similar to fluorescence confocal or 2-photon microscopy, the current USF system adopts a point-by-point scanning method so that the USF signal strength is directly correlated to the distribution of the contrast agent, although it is not directly equal; (3) the maximum scanning range along the depth direction in this study is 8.128 mm, which is relatively small and the errors caused by natural attenuation along the depth direction may be tolerable; (4) because USF imaging is still in the early development stage, adopting the current method is an efficient way to avoid complicating the methods and results, and more strict methods can be developed in future. As discussed before, the shapes of USF signals may be different according to the quantity of ICG-NPs around the ultrasound focus. It should be noticed that the three peak times in Figure S5(b) are slightly shifted, which are not in Figure 1(g). This is because the ICG-NPs may be well spread in the spleen, rather than only being confined in the silicone tube in which situation thermal diffusion would affect the shape of the USF signal less significantly.

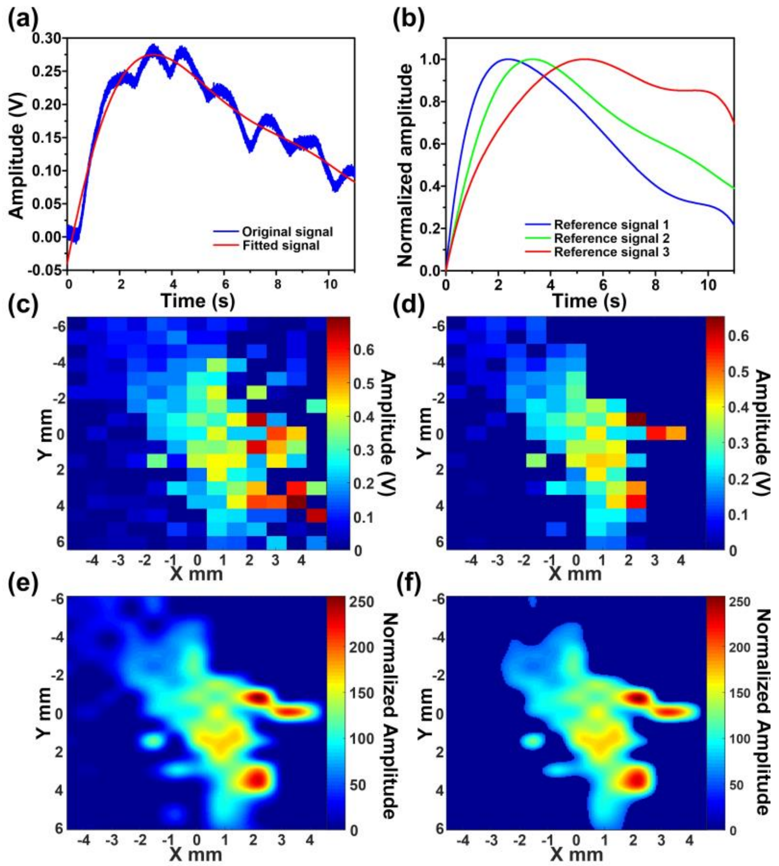

Figure S5. USF image processing. (a) Original (blue line) and fitted (red line) USF signal. (b)Three USF fitted curves used as three reference signals for correlation. USF images: (c) before correlation, (d) after correlation, (e) after interpolation based on (d), (f) after image segmentation based on (e).

The biodistribution of the ICG-NPs in mice. Mice (female, 7‒8 weeks, 17g) of strain BALB/C purchased from Jackson Laboratory (Bar Harbor, ME, USA) were used for this experiment. As shown in Figure S6, fluorescence imaging was carried out at various time points—i.e., 0, 1, 2, 4, 6, 8, 10, 24, 48, 72, 96, 144, 216, 336, 672 hours (h) after the intravenous (i.v.) injection of 120 μL ICG-NPs (173.65mg/kg). Figure S6(a) shows the left side of the mouse body. The region where the spleen was located emitted a strong fluorescence signal and reached the highest value at 4 h after the injection. The signal intensity decayed slowly from 6 to 72 h and was below the threshold (0.2) at 216 h. Figure S6(b) shows the right side of the mouse body. The region where the kidney and liver were located emitted a moderate fluorescence signal. The intensity stayed near the maximum value between 6 and 72 h and slowly decayed from 72 to 672 h at which time the intensity was below the threshold (0.125). Figure S6(c) shows the ventral side of the mouse body. The region where the liver was located emitted a moderate fluorescence signal. The intensity kept increasing slowly from 0 to 72 h and remained stable from 72 to 144 h. After that, the intensity decreased slowly and eventually reached the threshold (0.125) at 672 h. The signals emitted from the region of the liver and kidney had similar intensity but were much weaker than the signal from the spleen region.

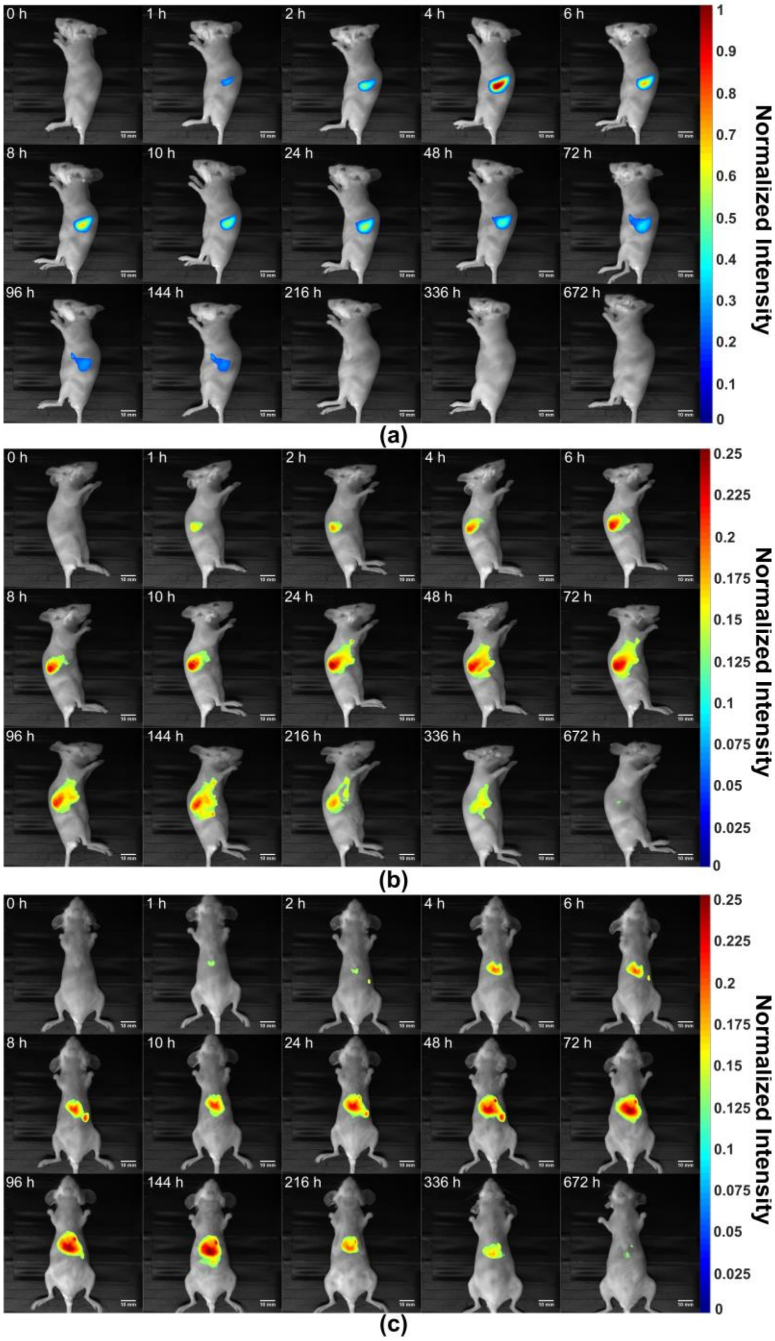

Figure S6. Fluorescence images of mouse body at different time points after i.v. injection of ICG-NPs (Ex/Em=808/830 nm) (a) left side; (b) right side; (c) ventral side.

Comparison of the anatomic locations of the mouse's spleen, liver, and kidney with the locations on the fluorescence images. To compare the anatomic locations of the mouse's spleen, liver, and kidney with the locations on the fluorescence images in Figure S6, the skin of the mouse was removed after sacrificing the mouse, and white-light photos were taken at the corresponding mouse positions. Figure S7(a) shows the photo taken from the left side of the mouse body. The spleen is indicated by a yellow solid line; the left kidney, a green solid line. Both are on the left side of the mouse body, close to the skin, and can be clearly seen. A part of the liver is indicated by a red solid line. This photo shows no significant spatial overlap among the spleen, liver, and left kidney. Because the fluorescence intensity from the liver and kidney are much weaker than from the spleen, it can be inferred that the fluorescence signal in Figure S6(a) mainly indicates the spleen. Figure S7(b) shows the photo taken from the right side of the mouse body. The right kidney is indicated by a green solid line, and a part of the liver by a red

solid line. Both are close to the skin of the right side of the body. Figure S7(c) shows the photo taken at the ventral side. The majority of the liver indicated by a red line is shown at the center of the body, and a small part of the spleen indicated by a yellow line can be seen at the right bottom of the liver. These anatomic locations in Figure S7 agree with the locations of the fluorescence image on the mouse body in Figure S6.

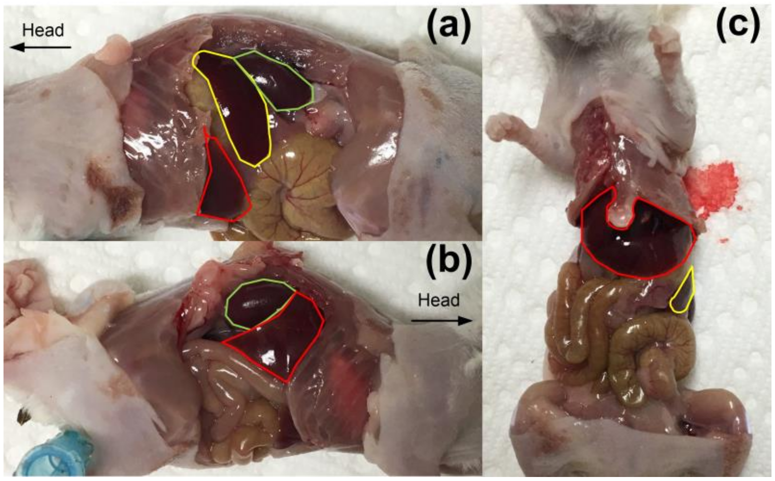

Figure S7. Locations of liver, kidney and spleen in the mice body. White-light photos of (a) left side; (b) right side; (c) ventral side of the mouse (spleen: yellow solid line, kidney: green solid line, liver: red solid line).

Comparison of USF imaging in living and dead mouse. A mouse (female, 9 weeks) of strain BALB/C purchased from Jackson Laboratory (Bar Harbor, ME, USA) was used for this experiment. The 70 µL mixed contrast agent (52.5 µL ICG-NPs with an LCST of ~35–36 °C and 17.5 µL ExiTron nano 12000) was locally injected into the mouse's right hind leg. Two USF scans were conducted at the same location in a mouse before and after her death (Figure S11(a)). Figure S8(a) shows the fluorescence 2D planar image (Ex/Em:808/830 nm) in the right hind leg where the contrast agent was injected and a white-light photo of the mouse is shown on the top right corner. The red box indicates the scan area of the USF imaging on the horizontal plane. An area of 5.08 × 5.08 mm^2 was raster scanned by the HIFU transducer with a driving voltage of 120 mV (~1.74 W of ultrasound power) and an exposure time of 400 ms. The light intensity illuminating the tissue was 1.39 mW/cm^2, which was measured by a power and energy meter (PM100D, Thorlabs Inc. Newton, NJ, USA). The lateral step size was 0.508 mm, and the axial step size was 1.27 mm. Figure S8(b) and S8(c) show the images of the body temperature before and after the death, respectively. The shell temperature of the living mouse's leg is ~34–35 °C while that of the dead mouse's leg is ~24–25 °C, which is far below the LCST of the ICG-NPs (~35–36 °C) used in this experiment. Thus, by using the same experimental parameters, a USF image was successfully acquired when the mouse was living (Figure S8(d)). In contrast, almost no USF signal was observed from the dead mouse (Figure S8(e)). This result shows the importance of matching the background temperature close to the temperature switching threshold (i.e., LCST of the contrast agent).

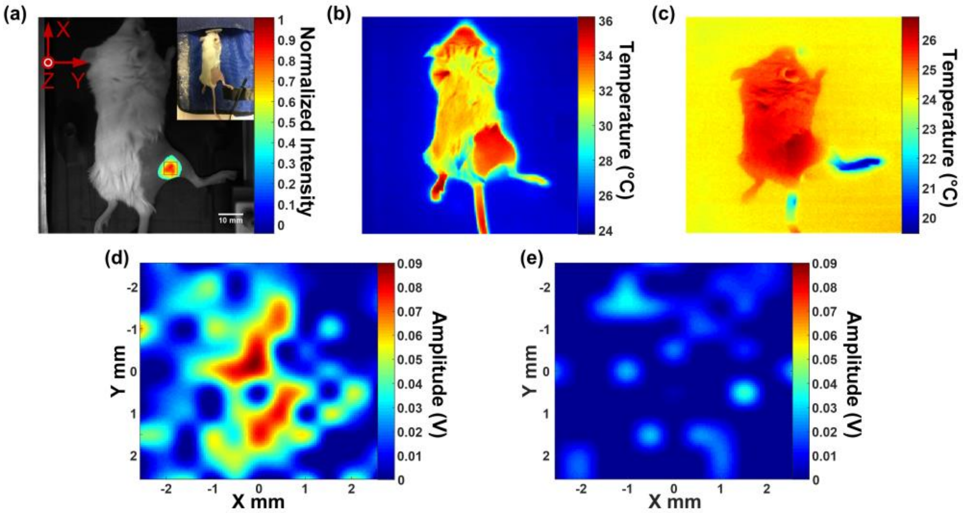

Figure S8. Comparison of USF imaging before and after the dead of a mouse. (a) A 2D planar fluorescence image (Ex/Em=808/830 nm) at the location of the injection on the mouse's right hind leg overlapped on a black-and-white photo. The USF scan area on the horizontal plane is indicated by a red square. A white-light photo of the mouse is shown on the upper right corner. The mouse body temperature images: (b) before and (c) after the death. 2D USF images (d) before and (e) after the death.

USF imaging system. The USF imaging system was similar to the one described in our previous studies[3]. A function generator (FG, 33220A, Agilent, Santa Clara, CA, USA) was used to modulate the intensity of the excitation laser (808 nm, MGL-II-808-2W, Dragon lasers, JL, China) into a 1 kHz sinusoidal wave and also provided a synchronized 1 kHz reference signal for the lock-in amplifier (LIA; SR830, Stanford Research Systems, Sunnyvale, CA, USA). The modulated light passed through a band-pass filter (FF01-785/62-25, Semrock Inc., Rochester, NY, USA) and illuminated the sample. The light intensity reached on the tissue's surface was 3.13 mW/cm^2 for porcine muscle tissue phantom, 1.48 mW/cm^2 for the porcine heart tissue phantom, 3.16 mW/cm^2 for the mouse leg, 4.87 mW/cm^2 for the mouse tumor, 2.87 mW/cm^2 for the spleen in the living mouse, and 3.67 mW/cm^2 for the *ex vivo* spleen in the porcine heart tissue phantom. These numbers are calculated based on the illumination area and the measured optical power (via a power and energy meter, PM100D, Thorlabs Inc. Newton, NJ, USA). The emitted fluorescence from the sample was collected by a fiber bundle and filtered by a set of emission filters, two long-pass filters (BLP01-830R, Semrock Inc., Rochester, NY, USA) and two absorptive filters (FSRRG830, Newport Corporation, Irvine, CA, USA). It was finally focused on a cooled photomultiplier tube (PMT, H7422-20 driven by a high-voltage source C8137-02, Hamamatsu, Japan) and converted into an electronic signal with a 1 kHz frequency. This 1 kHz signal was then amplified by a low-noise current preamplifier (SR570, Stanford Research Systems, Sunnyvale, CA, USA) and sent into the LIA. A HIFU transducer (2.5 MHz, H-108, Sonic Concepts Inc., Bothell, Washington, USA) driven by another function generator (FG, 33500B, Keysight Technologies, Santa Rosa, CA, USA) was used to switch on/off the USF contrast agent. The driving signal was amplified by a radio frequency power amplifier (RF-AMP, 325LA, E&I, Rochester, NY, USA). The amplified signal was delivered to a matching network (MNW) and then the HIFU transducer to generate an ultrasonic wave. The triggers from this function generator, which was working in burst mode, were used to control a data acquisition card (DAC, PCIE-6363, National Instruments, Austin, TX, USA) to sample the output signals from the LIA and the preamplifier. A motorized translation stage (Velmex Inc. Bloomfield, NY, USA) was used to realize the sample scanning. The temperature of the water was controlled by a temperature controller (PTC10, Stanford Research Systems, Sunnyvale, CA, USA) with a heater and a temperature sensor. A magnetic stirrer (11-100-16S, Fisher Scientific, USA) along with a long magnetic bar was used to make the water temperature uniform.

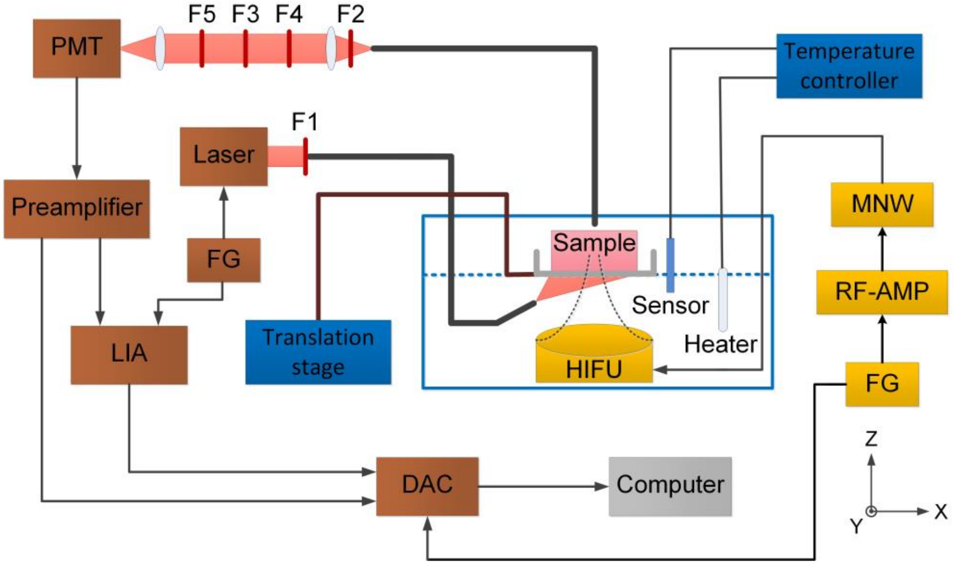

Figure S9. The schematic diagram of the USF imaging system. FG: function generator; RF-Amp: radio-frequency power amplifier; MNW: matching network; HIFU: high intensity focused ultrasound; PMT: photomultiplier tube; LIA: lock-in amplifier; F1: excitation filter (785/62 BP); F2-F3: emission filters (830 LP); F4-F5: emission filters (RG 830); DAC: data acquisition card.

Sample configuration of tissues. Figure S10 (a) and (b) show photos of the tube embedded porcine muscle tissue and the heart tissue, respectively.

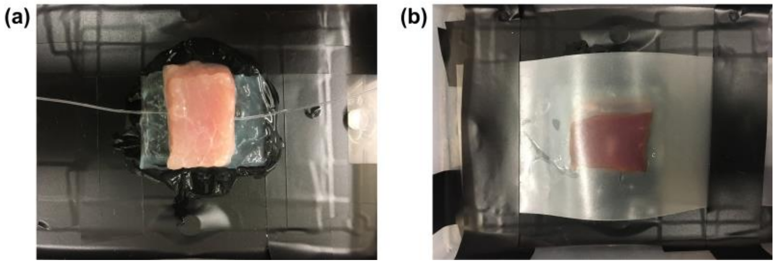

Figure S10. Sample configuration of tissues. (a) tube embedded porcine muscle tissue and (b) porcine heart tissue.

Experiment setup of *in vivo* USF imaging. Figure S11 shows the photos of the animal setup in the *in vivo* USF imaging. The HIFU beam was aligned with the photon collection fiber bundle. The focus of the HIFU was positioned at the bottom of the small tank before the experiment and was raised up in experiments. As shown in Figure S11(a) and (b), the excitation light illuminates the mouse leg or tumor from the bottom. As shown in Figure S11(c), the excitation light illuminates the mouse body from the top because the spleen is adjacent to the skin of the mouse left body.

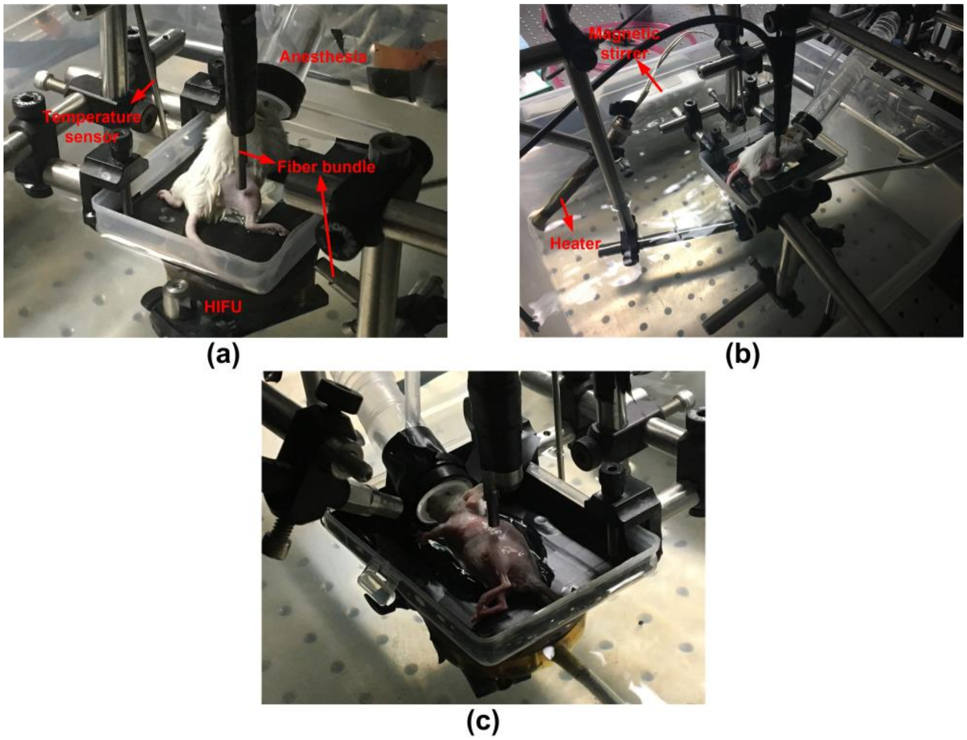

Figure S11. Photos to show the animal setup in the *in vivo* USF imaging experiments: (a) in the experiment of imaging mouse leg; (b) in the experiment of imaging mouse tumor; (c) in the experiment of imaging *in vivo* spleen after i.v. injection.

Table S1: A summary of HIFU driving voltage Vpp in this study. These numbers are from Figures 1, 2, 3, 4 and 6.

	**Tissue status (dead or live)**	**Applied HIFU driving voltage Vpp (mV)**
**Tube embedded porcine muscle tissue** (Figure 1)	Dead	As low as 60 mV (60, 80, 100, 120, 140, 160, 180)
**Porcine heart tissue** (Figure 2)	Dead	120
***In vivo* tumor** (Figure 3)	Live	250
***In vivo* spleen** (Figure 4)	Live	200
***Ex vivo* spleen** (Figure 6)	Dead	140

Reference

1. Cheng, B. *et al*. Development of Ultrasound-Switchable Fluorescence Imaging Contrast Agents Based on Thermosensitive Polymers and Nanoparticles. *IEEE J. Sel. Top. Quantum Electron.* **20**, 67-80 (2014).
2. Liu. J. & Li. W. The Automatic thresholding of gray-level pictures via two-dimensional otsu method. *Acta Automat. Sinica* **1**, 015 (1993).
3. Cheng, B. *et al.* High-resolution ultrasound-switchable fluorescence imaging in centimeter-deep tissue phantoms with high signal-to-noise ratio and high sensitivity via novel contrast agents. *PloS one* **11**, e0165963 (2016).
